# Sustain, Adapt, and Overcome—Hypoxia Associated Changes in the Progression of Lymphatic Neoplasia

**DOI:** 10.3389/fonc.2019.01277

**Published:** 2019-11-21

**Authors:** Orsolya Matolay, Gábor Méhes

**Affiliations:** Department of Pathology, Faculty of Medicine, University of Debrecen, Debrecen, Hungary

**Keywords:** hypoxia, lymphoma, cancer, necrosis, metabolic adaptation

## Abstract

Irregular perfusion and related tissue hypoxia is a common feature of solid tumors the role of which in the survival and progression cancer has been gradually recognized. Adaptation and selection mechanisms in hypoxic areas in solid tumors are regulated by Hypoxia Inducible transcriptional factor 1 (HIF1) and other hypoxia mediators and are associated with aggressive clinical behavior in a large spectrum of malignancies. Aggressive forms of lymphatic neoplasias present with solid tumor-like features, also including rapid cell growth, necrosis and angiogenesis, the clinical potential of which is still underestimated. While the role of regional hypoxia in normal B-cell maturation and malignant transformation is becoming evident, the impact of tissue hypoxia on their behavior is not well-understood. Compared to some of the common solid cancer types data for some of the key regulators, such as HIF1 and HIF2, and for their downstream effectors are available in a limited fashion. In the current review we aim to overview the physiological aspects of major hypoxia pathways during B-cell maturation and adaptation-related changes reported in lymphatic neoplasia covering important targets, such as carbonic anhydrases IX and XII (CAIX, CAXII), glucose transporter 1 (GLUT-1) and vascular endothelial growth factor (VEGF). In conclusion, experimental and clinical results direct to important but currently unexploited role of hypoxia-driven resistance mechanisms especially in aggressive forms of B-cell neoplasia.

## Introduction

The general presentation and clinical behavior of malignant neoplasias are strongly dependent on tissue oxygenation and nutrient supply. Lymphomas are consisted of transformed cell masses with at least some circulating capacity, and thus, have long been believed to be independent on tissue blood perfusion. Indeed, the limited metabolic needs of indolent lymphoproliferations featured by low proliferative activity may be directly served from the circulation. However, high-grade variants growing in a solid tumor-like fashion require continuously growing perfusion in parallel with the progressive increase of the tumor mass. Large tumor burden and related intra-tumoral pressure dispose to relative tissue perfusion deficit similar to other cancer types. Significant oxygen depletion will result in hypoxic areas turning into focal necrosis frequently seen in aggressive large cell lymphomas (1), and in the progressive variants of Hodgkin's lymphoma (2).

Hypoperfusion may be transient or may occur in a moderate fashion. Sublethal forms of hypoxia in cancer were associated with metabolic reprogramming and adaptation leading to partial tumor resistance and recovery (3). The clinical behavior and the therapeutic response was found to be dependent on the O_2_ supply of the tumor tissue and the hypoxic tumor mass could be related with adverse prognosis in diverse malignancies (4). However, the general impact of tissue hypoxia in lymphomas remains unknown. As compensatory modifications potentially interact with aggressive phenotype and therapeutic resistance, we felt the need to overview and summarize the available knowledge on hypoxia-associated functional changes reported in both normal and neoplastic lymphoproliferations.

## Hypoxia and HIFs

Tissue hypoxia is the result of the imbalance of local O_2_ consumption and supply due to several reasons, including increased cellular activity or reduced perfusion. The consequence of hypoxic stress at the cellular level is the activation of adaptation mechanisms, and the key regulators are the Hypoxia Inducible transcriptional Factors (HIFs). Oxygen and energy dependence can be managed by the immediate stimulation of glucose uptake and utilization in physiological and pathological conditions. As a remarkable integral feature, transformed cells perform elevated glycolytic activity despite sufficient O_2_-levels (the Warburg-effect), representing dual capacity for glycolytic and oxidative metabolism (5, 6). Although the exact clinical role of this alternating carbon substrate utilization is still unclear, metabolic heterogeneity offers high-grade flexibility and selective advantage in a hypoxia independent fashion (7, 8).

The adaptive mechanisms regulated by the HIFs are the major pathways induced in response to hypoxia and are recently also considered as potential therapeutic targets (9–12). The active HIF-heterodimer complexes are consisted of α and β subunits (13–15). The oxygen-sensitive α subunit has three isoforms (HIF-1α, HIF-2α, HIF-3α), while the β subunit is expressed constitutively in an oxygen-independent manner.

**Figure 1:**
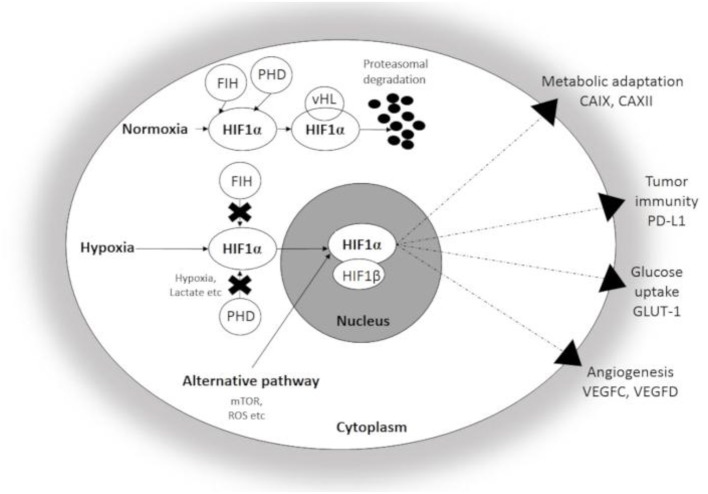
Schematic illustration of Hypoxia Inducible factor 1-α (HIF1α) associated changes in hypoxic conditions. Under normoxia, HIF1α undergoes rapid proteasomal degradation due to the oxygen-dependent modification by PHDs. In contrast, HIF1α is stabilized and translocated into the nucleus under hypoxia, and after dimerization with the β-subunit, the active heterodimer initiates the transcription of hypoxia-responsive genes participating in adaptation. CAIX, CAXII, carbonic anhydrase IX and XII; GLUT-1, Glucose transporter 1; PD-L1, Programmed cell death ligand 1; VEGFC and D, Vascular Endothelial Growth Factor C and D; FIH, Factor Inhibiting HIF; PHD, Prolyl hydroxylase.

HIF1α, a master regulator of cellular adaptation, has been widely studied in different experimental and pathological conditions (Figure 1). Under normoxia, HIF-1α is hydroxylated by prolyl hydroxylases (PHDs) and binds to the von Hippel-Lindau (vHL)-protein which leads to the ubiquitin-proteasomal degradation of the molecule. However, PHDs are largely inactivated by hypoxia and HIF1α enters the nucleus and binds the β subunit (8, 12, 13). The active complex induces the transcription of a set of genes associated with cellular adaptation. Thus, hypoxia promotes metabolic reprogramming and survival in both normal and transformed cells and thus, plays an important role in the resistance to chemo- and radiotherapy (7, 13, 16, 17). Similar to HIF-1α, HIF-2α is an alternative heterodimer factor, the transcriptional activity of which is more prolonged and synchronizes a chronic response to hypoxia. Interestingly, the expression of HIF-2α is essential for the regulation of proinflammatory cytokines during macrophage activation and an effect on vascular remodeling was also reported. HIF-2α was also associated with unfavorable prognosis, poor overall and disease-free survival, progression and metastasis formation in solid tumors (18). HIF-3α is expressed by diverse human organs (kidney, heart, and brain) with the highest intensity levels during fetal organogenesis (19). HIF-3α seems to be closely interactive with HIF-1α and HIF-2α and controls gene expression in a tissue-specific manner, e.g., during lung development (20–22).

## Molecular Pathways Regulated by HIF-1α

The gene regulatory effect of HIF-1α and to a lesser degree of HIF-2α and HIF-3α following hypoxia was demonstrated at many levels, including the management of intracellular acidosis, angioneogenesis, glucose uptake, and metabolism.

Carbonic anhydrases are the members of a large zinc-metalloenzyme family which actively contributes to the pH adaptation of the cells. The role of these cell membrane-bound enzyme is to catalyze H^+^-capture and elimination in order to neutralize intracytoplasmic acidosis resulting from increased glucose utilization and lactate production. Normalization of the intracellular pH is associated with enhanced cell survival and the consequential extracellular acidosis -modifies the immediate microenvironment. CAIX and CAXII isoenzymes are considered as cancer-related adaptive factors and their upregulation was reported to be highly HIF1α-dependent. The characteristic expression of CAIX is strongly associated with intratumoral hypoxia and can be nicely studied in histological conditions. The expression of CAIX and CAXII correlated with chemo- and radio-therapy resistance in a large variety of cancer types (7, 23, 24).

Glucose transporter type 1 (GLUT1) expression is often increased in malignant tumors characterized by high glycolytic rate and intense energy consumption. The overexpression of GLUT1 was found to be associated with the poor prognosis of several major tumor types, including colon and breast carcinoma (25–29).

Vascular endothelial growth factor (VEGF) is a member of a multifunctional group of cytokines which stimulates neoangiogenesis both in physiological and pathological conditions. The family consists of five members: VEGFA, VEGFB, VEGFC, VEGFD, and placenta growth factor (PIGF). The effect is transduced through tyrosine-kinase receptors VEGFR-1 and VEGFR-2 (30–32). VEGFR-3 is a unique receptor binding the ligands VEGFC and VEGFD. VEGFC and VEGFD are expressed by lymphatic endothelial cells playing an important role in lymphangiogenesis (33–35).

## Tissue Oxygenation and Hypoxic Stress in Physiological B-Cell Maturation

The availability of oxygen in normal lymphatic tissues is highly variable and this potentially affects normal immune functions according to the results of *in vivo* animal experiments. The distance measured between the blood vessels and germinal centers (GC) within lymphatic tissues was consequently larger than expected, the majority of the GCs were >40 μm away from the nearest vascular structure in murine spleen (36). The results indicated that the GCs are preferentially sited in hypoxic areas. Furthermore, GL-7, a marker of the GC B-cells was upregulated in these hypoxic B-cell areas, while HIF production was also increased compared to B-cells in normoxic areas (36). Similar variations of the local oxygen pressure within the bone marrow was described in an alternative *in vivo* mouse model (37) and it was found that the perisinusoidal parenchyma, approximately 40 μm away from the endosteum, was far less oxygenated and showed significant vascular irregularity compared with the peritrabecular zone.

Both the maturation and activation of the normal lymphatic cells seem to be influenced by the actual oxygen supply. The HIF-1α-regulated glycolytic pathway essentially contributed to the survival and the differentiation of the B-cells and was found especially critical for the transition from the pro-B to pre-B cell stage (38). Furthermore, HIF-1α deficiency significantly influenced late B-lymphocyte responses (39). HIF-1α appears to be important for the development and the activation of B-cells and the resulting increase of the glycolytic rate essential for the initial maturation process. However, it is not clear how far HIF-1α plays a role in the terminal differentiation of the B-cells (40, 41). While HIF-1α is expressed in B-cells during physiological immune activation, HIF-2α overexpression was observed only following malignant transformation, e.g., in Diffuse large B cell lymphoma (DLBCL) (42).

Nevertheless, hypoxia appeared to play a dominant role follicular B-cell maturation (36). Hypoxic microenvironment was proposed to promote IgM to IgG class switch recombination (CSR). In *in vitro* conditions, the peak of the CSR kinetics was reached on the third day of experimental hypoxia that was followed by a significant increase of apoptotic caspase 3 activation. Accordingly, clonal competition and affinity maturation is influenced by the hypoxic niche of the follicular GC (36). The antibody composition in the hypoxic GC was investigated and the PHD/vHL/HIF axis was found to have a basic influence on the quality of antibody response. In particular, hypoxia had a direct effect on *activation induced deaminase* (AID) enzyme levels essential for the rate of ongoing CSR (43).

To accurately reflect the regulatory complexity it is important mentioning that oxygen independent activation of HIFs in immune cell maturation was also repeatedly suggested. The interaction of interleukins with the HIF-pathway in inflammation could be demonstrated, e.g., IL-4 induced HIF-2α, while IL-2 induced HIF-1α activation in CD8+ T-cells. In contrast, stabilization of HIF in B-cells is more likely to be associated with chemokine and Toll-like receptor, as well as through B-cell receptor signaling pathways (40).

## Hypoxia and Related Changes in NHL

### Intratumor Hypoxia and Adaptive Mechanisms in Non-Hodgkin B-Cell Neoplasias

B-cell lymphomas are biologically highly heterogeneous tumors. The earliest data on the metabolic setup were provided on DLBCL, the most common type of aggressive lymphomas of the adults. According to the studies applying genome-wide arrays and multiple gene expression clustering separate functional groups were established (44). DLBCL could be classified by the activity of (i) the B-cell receptor (BCR) signaling, (ii) the inflammatory/host response genes, and of (iii) the mitochondrial oxidative phosphorylation (OxPhos) genes. The OxPhos signature is highlighted by the increased expression of the mitochondrial respiratory chain complexes and other members of the electron transfer machinery (45, 46). As such, this special form of the Warburg-effect, mentioned earlier, represents a unique, BCR-independent intracellular stimulus within the clinical category of DLBCL.

Relative hypoxia and resultant intracellular acidosis in lymphoma are both expected to induce escape mechanisms through cellular adaptation similar to non-hematological solid cancer. Histological and experimental *in vitro* studies primarily concentrated on HIF-1-related reprogramming and on angioneogenesis as major components of hypoxia-related adaptation.

In cultivated lymphoma cells, constitutive HIF-1α activity and vHL suppression were noted resulting in the maintenance of the cancer stem cell phenotype (47). Not only were the cellular functions studied but also the effect of HIF1α in the context of oncogenic viral infection. Gene expression levels of Kaposi sarcoma-associated herpesvirus (KSHV) was investigated and HIF1α was found to contribute to the expression of the KSHV-encoded genes. In return, KSHV had a positive effect on the HIF1α levels suggesting a synergistic activation on each other (48).

Angiogenesis has been intensively studied in non-Hodgkin lymphomas (NHL). Minoia et al. examined neovascularization by CD34 immunostaining and HIF1α expression in biopsies of NHL patients. In paired diagnostic and second biopsies they observed the up-regulation of HIF1α and increased tumor vascularization in the follow-up biopsy which was explained by treatment-related stress adaptation mechanisms (49). A general increase in microvessel density (MVD) was described in NHL and classical Hodgkin's lymphoma (cHL) cases (34). A similar observation was reported demonstrating prominent angiogenesis in aggressive subtypes of NHL in parallel with increased MVD and inflammatory infiltrates. An active secretion of angiogenic cytokines by the neoplastic cells was proposed, which was supported by the elevated levels of soluble angiogenic factors measured in the sera of the subjects.

Further, angiogenic factors were also examined in NHL (50). The high expression of VEGFC correlated with aggressive features and short survival. VEGFA/VEGFC double negative cases had longer survival rates compared to the double positive cases. In support of these data, a meta-analysis concluded that VEGF isoforms offer a promising tool to predict outcome in high-grade lymphoma (51).

Hypoxia-related adaptive changes were recently demonstrated in B-cell lymphoma cells. *In vitro* response to hypoxia resulted in a highly dynamic fashion (e.g., CAIX upregulation) (52). HIF-1α, HIF-2α, VEGF, and CAIX expression were evaluated in clinical NHL samples as well (42). HIF-1α presented predominantly with nuclear localization within the lymphoma cells, while the HIF-2α was more prominent in adjacent reactive macrophages. Cell membrane CAIX was variably expressed mostly within the perinecrotic areas of DLBCL tissue samples. While the clinical impact of hypoxia and angiogenesis-related genes e.g., HIF-1α, VEGF, GLUT-1 is still not cleared, these genes are supposed to be widely upregulated, as also stated in a primary central nervous system DLBCL xenograft model (53).

The exact effect of hypoxia-associated markers on therapeutic response in B-cell malignancies also requires further clinical evaluation. Chemoresistance was associated with the parallel upregulation of the anti-apoptotic Bcl-_xL_ and HIF-1α and a study reported a positive correlation between these factors and the therapeutic efficacy in NHL cell lines (54). Furthermore, HIF-1α was shown to be a promising prognostic factor in DLBCL patients who received rituximab treatment. It was proposed that further to HIF-1α the CD20 antigen expression could also be induced by reactive oxygen species (ROS), thus, higher HIF-1α and CD20 levels together lead to increased therapeutic effect (55). Another study reported increased adaptive glucose metabolism and specifically hexokinase 2 (HK2) overexpression suggesting a potential therapeutic target in DLBCL (56).

Radiological signs of hypoxia and focal necrosis are prominent and well known in everyday lymphoma diagnostics and follow up. The FDG-PET-CT approach highlights the metabolic activity of the malignant tissue by applying glucose transporters as *in vivo* targets. In a clinical study on various lymphoma subtypes the direct correlation between 18F-FDG PET activity and tissue expression of GLUT1 but not of GLUT3 could be established (57). More recent data further support the potential clinical significance of the intratumoral metabolic status reflected by GLUT1 in aggressive lymphoproliferations (58, 59). Functional *in vivo* imaging of the hypoxic/necrotic tumor mass promises new perspectives for treatment decision.

### Hypoxia-Associated Changes in cHLs

The potential role of hypoxia in the evolution and progression of cHL was not in the spotlight in the past. Hypoxia-related pathways were not reported to be generally activated in HRS cells determined by gene expression (60) or epigenetic profiling (61, 62). On the other hand, a few data support the significance of hypoxia in the progression of cHL. A dynamic B-cell reprogramming and HRS cell phenotype switch induced by HIF-1a expression with the involvement of NFkB and MYC was suggested by Wein et al. (63). Moreover, basic changes in cellular metabolism and response to chemotherapeutic agents could be recently observed upon hypoxia mimetic treatment *in vitro* in Hodgkin lymphoma cell lines. Upregulation of selected hypoxia/metabolism-associated genes were observed, e.g., GLUT1 as well as therapy resistance genes, such as the antiapoptotic XIAP-associated factor 1 (XAF1) (64).

HRS cells are likely to be placed in niches with limited nutrient and oxygen supply and may be exposed to adaptation signaling in order to survive. Prolyl hydroxylase domain proteins (PHD1, PHD2, PHD3) were reported to have a role in the cellular oxygen homeostasis, moreover the expression of PHD1 and PHD3 (along with HIF1α and HIF2α) were associated with treatment resistance (65). Not only HIF-1α but HIF-2α has been studied in comparison with GLUT1 and Programmed cell Death Ligand 1 (PD-L1) (66). Interestingly, GLUT1 positivity had a prognostic significance in advanced cHL, and expression of GLUT1 significantly correlated with PD-L1 expression. The analysis of pro-angiogenic expression profile (VEGF, Ang-1, Ang-2, Tie-2) revealed a correlation between PD-L1 and VEGF, with an elevated MVD in the positive samples. However, tissue perfusion related markers did not show significant prognostic effect in this series of cHL cases (21, 22).

HIF-1α, VEGF, and platelet-derived growth factor receptor α (PDGFRα) were shown to be expressed in the HRS-cells, although only a limited correlation could be seen (67). A retrospective study focused on microvessel caliber in 286 cHL tissue samples where the diameter of the blood vessels correlated with the stage of the lymphoma. Angiogenetic adaptation was considered as an early event in cHL with the assumption that newly formed vessels were subject of easy penetration by the malignant cells (68). VEGFD expression was examined in cHL and a correlation was reported between the VEGFD and the elevated number of tumor microvessels (69). Doussis-Anagnostopoulou et al. studied VEGF expression in HRS-cells and described diffuse cytoplasmic and/or focal paranuclear staining both in the HRS-cells and in the bystanding reactive component. The production of VEGF was also investigated in different cHL cell lines. VEGF promoted monocyte chemotaxis and inhibited the maturation of the antigen-presenting dendritic cells. In their interpretation, VEGF promotes neoplastic progression through the inhibition of the anti-neoplastic immune effector functions (70). Studies on cHL cell lines, cHL lymphoma tissue, and on inflammatory lymphs nodes revealed the overexpression of VEGFC in HRS-cells promoting lymphangiogenesis through a VEGF3-R receptor-associated pathway. Correlation of the results with clinical data suggested higher risk for treatment failure and recurrence upon elevated VEGFC expression rates (71).

Experimental and clinical data are also limited regarding adaptation mechanisms to hypoxia-derived intracellular acidosis. However, recent data show the hypoxia-associated phenotype switch strongly influences therapeutic success in cHL, similar to other malignancies. In a recent work by us (2) highly specific cell membrane CAIX expression could be demonstrated in HRS-cells in the 45% of evaluated cHL cases, which showed a positive correlation with tissue necrosis determined histologically. Focal CAIX expression in HRS cells was associated with depressed cell proliferation capacity determined by Ki-67 nuclear positivity. CAIX expression could be measured by digital image analysis and proved to be different between the major histological subtypes of cHL (72). Moreover, cHL cases featuring selective CAIX-expression in the initial diagnostic sample showed a significantly decreased progression-free survival compared to the CAIX-negative cohort (2).

## Concluding Remarks

Despite the increasing amount of experimental and clinical data it is only clear that hypoxia related effects in lymphoproliferative neoplasias are highly complex and heterogeneous, clinical relations remain largely unknown. In contrast to the initial skepticism, numerous details support the specific role of metabolic adaptation in therapy response and immune accessibility. The relation between perfusional/nutritional defects and associated effectors seems to be established, however, tissue biomarkers with the clear clinical impact are still in the validation phase. More focused studies are needed to evaluate the exact significance of HIF-1/2α mediated downstream effectors, such as CAIX, CAXII, GLUT-1, VEGF in a cell of origin context. Moreover, the correlation of tissue-based and related *in vivo* metabolic imaging findings may better define the actual role and the dynamics of hypoxia during lymphoma management.

## Author Contributions

All authors listed have made a substantial, direct and intellectual contribution to the work, and approved it for publication.

### Conflict of Interest

The authors declare that the research was conducted in the absence of any commercial or financial relationships that could be construed as a potential conflict of interest.

## Abbreviations

AID: activation induced deaminase
CAIX: carbonic anhydrase IX
CAXII: carbonic anhydrase XII
cHL: classical Hodgkin lymphoma
CSR: class switch recombination
DLCBL: diffuse largeB cell lymphoma
GC: germinal center
GLUT-1: Glucose transporter type 1
HK2: Hexokinase 2
HIF: Hypoxia Inducible transcriptional factor
HRS: Hodgkin-sternberg-reed cell
KSHV: kaposi sarcoma-associated herpes virus
MVD: microvessel density
NHL: non-Hodgkin lymphoma
PD-L1: programmed cell death ligand-1
PDGFRα: platelet derived growth factor receptor α
PHD: prolyl hydroxylase
ROS: reactive oxygen species
VEGF: vascular endothelial growth factor
vHL: von Hippel Lindau
XAF1: XIAP-associated factor 1.
